# Evaluation of the QUANTUM BLUE sCAL rapid test as a point of care tool to identify patients with peritonsillar abscess

**DOI:** 10.1038/s41598-021-84027-w

**Published:** 2021-02-24

**Authors:** Lea-Sophie Stahl, Johannes Roth, Claudia Rudack, Annika McNally, Jakob Weber, Thomas Vogl, Christoph Spiekermann

**Affiliations:** 1 Department of Otorhinolaryngology, Head and Neck Surgery, University Hospital Münster, 48149 Münster, Germany; 2 Institute of Immunology, University Hospital Münster, Röntgenstr. 21, 48149 Münster, Germany; 3 BÜHLMANN Laboratories AG, 4124 Schönenbuch, Switzerland

**Keywords:** Diseases, Oral diseases, Biomarkers, Diagnostic markers, Medical research, Biomarkers

## Abstract

S100A8/A9 (Calprotectin) serves as a biomarker for various inflammatory diseases, such as for peritonsillar abscess (PTA). Recently, the PTA score was developed for reliable PTA identification. It uses a combination of characteristic clinical symptoms and elevated calprotectin levels in serum and saliva to determine this score. Although well-established point-of-care tests (POCT) to determine serum or faecal calprotectin levels exist, a reliable and rapid tool to analyse salivary calprotectin has not yet been described. In this study, we analysed the potential of the QUANTUM BLUE sCAL Test (QBT, BÜHLMANN Laboratories AG, Switzerland) to determine S100A8/A9 levels during outpatient management. These QBT measurements are combined with other clinical factors to determine the PTA score. Significantly higher calprotectin levels were determined by QBT in patients with PTA compared to healthy controls. The receiver operating characteristic (ROC) curves for the QBT revealed cut-off values of 2940 ng/ml (sensitivity = 0.88, specificity = 0.78) in serum and 5310 ng/ml (sensitivity = 0.80, specificity = 0.50) in saliva. By adding the QBT results to determine PTA values, a ROC analysis provided a statistical cut-off score of 2.5 points to identify the existence of a PTA with a sensitivity of 100% and a specificity of 89.3%. The QUANTUM BLUE sCAL Test (QBT) is an appropriate POCT to determine serum and salivary calprotectin levels. Thus, PTA scores can be determined within a short time frame by applying the QBT during outpatient management.

## Introduction

The myeloid-related proteins S100A8 (MRP8) and S100A9 (MRP14) belong to the S100-protein family and form a calcium-binding heterodimer, calprotectin (S100A8/A9), under physiological conditions^1–3^. S100A8 and S100A9 are considered to be danger associated molecular patterns (DAMPs), as they are released predominantly by neutrophils and monocytes during inflammatory processes, and have the potential to further activate and recruit leukocytes via the Toll-like Receptor 4 (TLR-4) and via the receptor of advanced glycation end products (RAGE) pathway^3–6^.

Increased levels of calprotectin have been found in several diseases, such as in rheumatoid arthritis, inflammatory bowel disease or cardiovascular diseases, indicating to be a more sensitive biomarker for diagnosis and monitoring of disease activity than C-reactive protein (CRP) or erythrocyte sedimentation rate (ESR)^7–13^.

Recently, we could observe increased calprotectin levels in saliva and serum of patients suffering from peritonsillar abscess (PTA)^14^. The PTA is a common but also grave infection as it can cause life-threatening complications^14,15^. Hence, early diagnosis of PTA and initiation of an appropriate treatment approach is essential to avoid potential clinical complications, such as airway obstruction or pneumonia^16,17^. To facilitate early diagnosis, the PTA score was developed by taking characteristic clinical symptoms of PTA and elevated S100A8/A9 levels in saliva and serum into account. This PTA score represents the first instrument that allows for a reliable diagnosis and differentiation between PTA and the less severe form, peritonsillar cellulitis (PC)^14^. Furthermore, an adequate treatment approach can be determined after the application of this novel PTA score, therefore avoiding unnecessary surgical interventions^18^.

The enzyme-linked immunosorbent assay (ELISA) represents the current “gold standard” for measuring S100A8/A9 levels and is also a well-established method. However, this methodology is highly time-consuming and, thus, not an appropriate tool for rapid outpatient management. The QUANTUM BLUE sCAL Test (QBT, BÜHLMANN Laboratories AG, Switzerland), on the other hand, is designed as a point-of-care testing (POCT) method for quick and simple measurement of calprotectin in serum (sCAL). This rapid test not only shows a good correlation to ELISA measurements, but is also very practical and appears more patient-friendly, since several traumatic invasive treatments can be avoided due to its fast and quantitative results on-site^19,20^. Until now, various studies have used ELISA tests and some have even used the QBT to determine calprotectin levels in serum, faecal samples or synovial fluid in cases of various inflammatory diseases^9,13,20–24^. However, the potential of the QBT as a point-of-care tool to determine S100A8/A9 levels in saliva samples has not yet been described and no other point-of-care device currently exists for analysing salivary calprotectin levels. Due to the reduction of invasive medical examination and its simple non-expert handling, the QBT can be considered as a suitable alternative to ELISA measurements for outpatient care. Therefore, it was the aim of the present study to analyse the potential of the QBT to quickly determine salivary S100A8/A9 levels during outpatient consultation and to determine its applicability in the diagnosis of peritonsillar abscess by using the PTA score.

## Results

### Patients and selection of the samples

Patients (n = 179) with a median age of 24 (range 2–83 years) were included in the present study. The study population consisted of 84 male and 95 female patients (male-to-female ratio of 0.88). The patients showed various tonsil-related diseases, such as tonsil hyperplasia (n = 16; median age 6 years; range 2–32 years), recurrent tonsillitis (n = 71; median age 24 years; range 7–59 years), acute tonsillitis (n = 15; median age 25 years, range 13–65 years), mononucleosis (n = 10; median age 24.5 years; range 18–59 years), peritonsillar abscess (n = 36; median age 32.5 years; range 10–83 years), and peritonsillitis (n = 16; median age 27.5 years; range 7–66 years). Healthy volunteers (n = 15; median age 30 years; range 26–59 years) without any history of tonsillitis or tonsil-related diseases served as controls.

### QUANTUM BLUE sCAL rapid test to determine salivary calprotectin levels

**Figure 1:**
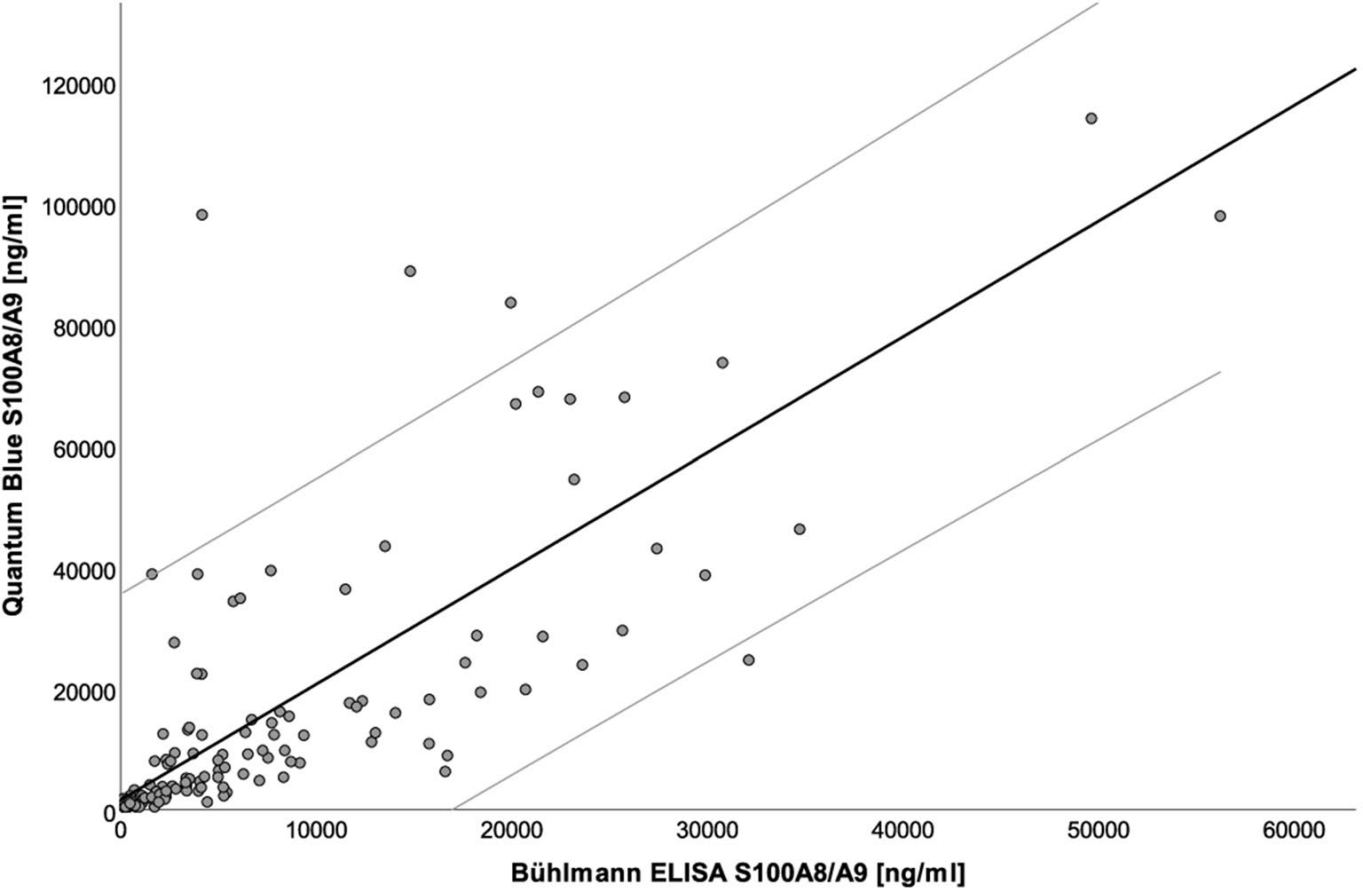
Calprotectin levels in saliva. Scatter plot showing the correlation between calprotectin levels in saliva determined by QUANTUM BLUE sCAL rapid test and the BÜHLMANN MRP8/14 ELISA with a Spearman correlation of r_sp_ = 0.848 (black line, p < 0.001) and the 95% confidence interval (95% CI 0.245; 0.336, grey lines).

**Figure 2:**
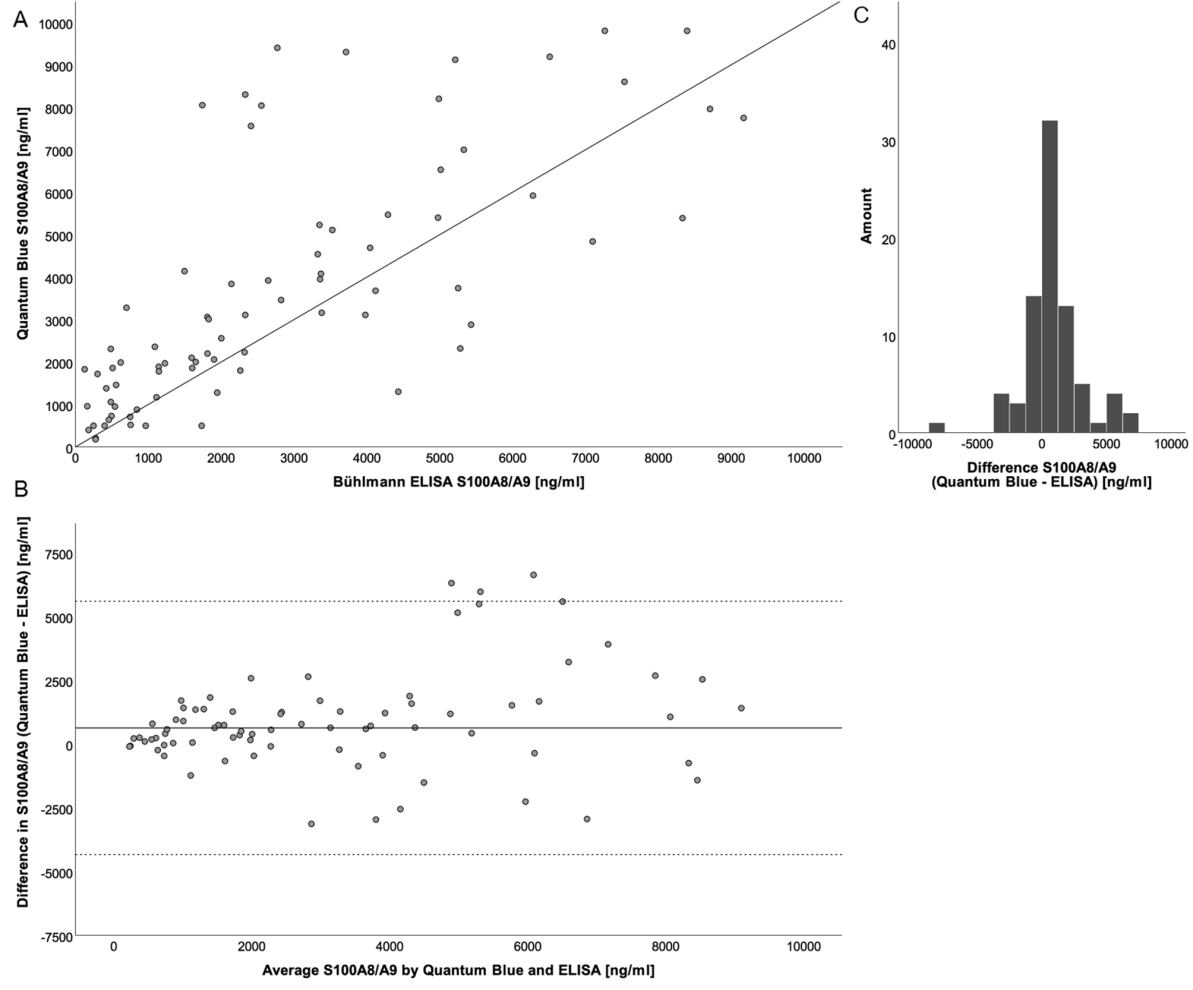
Agreement between QUANTUM BLUE and ELISA. Scatter plot of S100A8/A9 concentration in saliva determined by ELISA and QUANTUM BLUE rapid test with line of equality (f(x) = 1 × x + 0) to illustrate the degree of agreement (**A**). The Bland–Altman plot of difference between S100A8/A9 levels determined by QUANTUM BLUE and ELISA against mean S100A8/A9 levels shows a positive bias for the QBT (solid line, mean; dashed line, 2 × standard deviation; (**B**)). The differences follow a normal distribution, which is illustrated by a histogram (**C**).

The QBT rapid test was developed and validated for the determination of serum calprotectin levels^25^. Therefore, we had to prove whether the QBT was suitable to determine salivary calprotectin levels as well. Excellent significant, positive correlations between calprotectin levels measured by QBT and ELISA could be observed in saliva (r_sp_ = 0.848, regression ß = 0.290, 95% CI 0.245; 0.336, p < 0.001) (Fig. 1). In order to check for potential deviations between the two types of measurement, we performed a Bland–Altman analysis^26^ and have found a slightly positive bias for QBT levels as compared to ELISA for samples measuring in the clinically relevant range of ≤ 12000 ng/ml (Fig. 2A–C). These data demonstrate that there may be a need to determine revised cut-off values to use the QBT in PTA score determination.

### Calprotectin levels in patients with peritonsillar abscess

**Figure 3:**
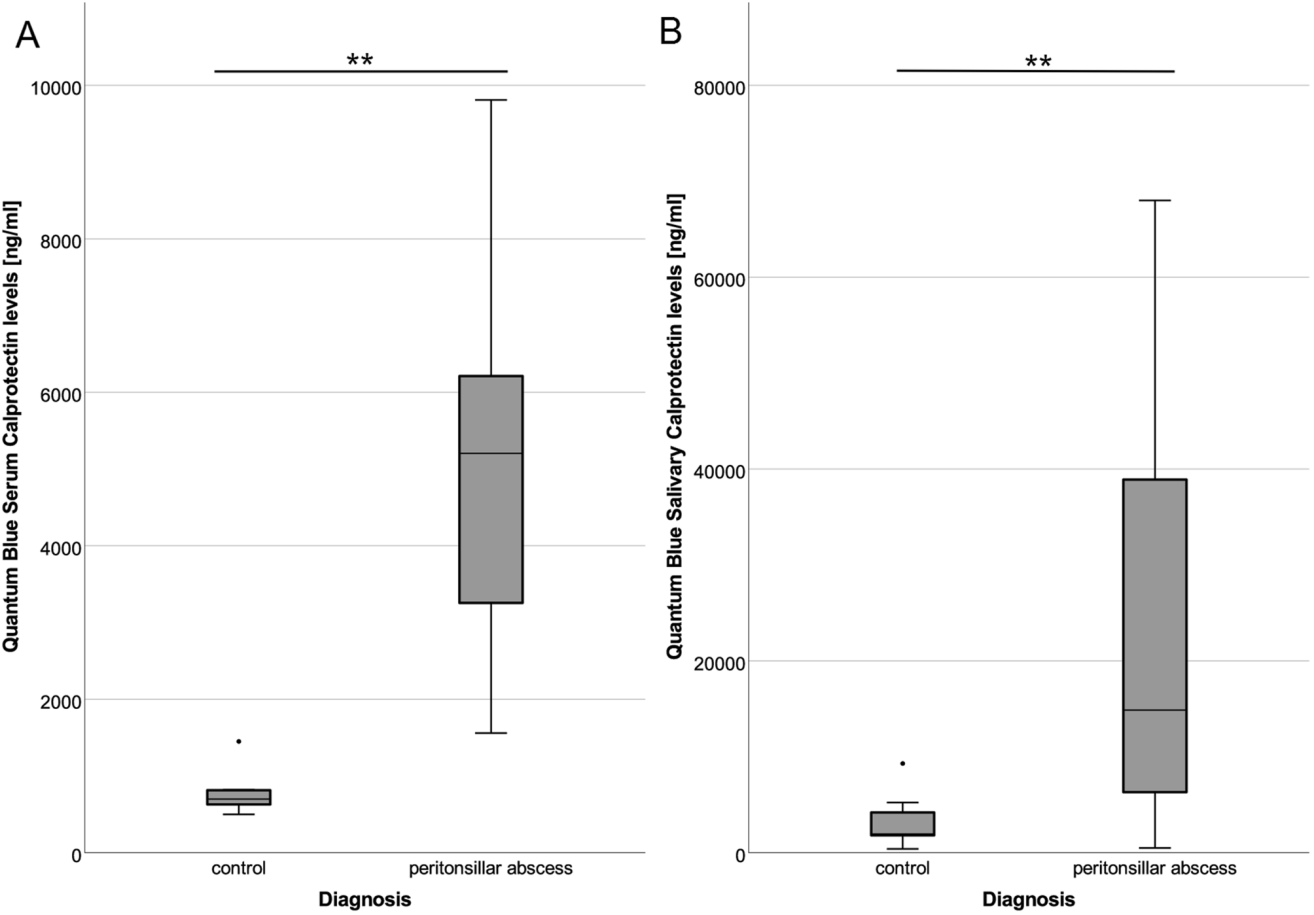
Calprotectin levels in patients with peritonsillar abscess. Significantly increased levels of calprotectin were determined by QUANTUM BLUE sCAL rapid test in patients with peritonsillar abscess compared to healthy controls in serum (**A**) and saliva (**B**) (***p < 0.001, **p < 0.01).

Compared to controls, significantly increased levels of calprotectin are observed in serum (5745 ± 828 ng/ml vs. 780 ± 103 ng/ml, p < 0.001) and saliva (25,825 ± 5943 ng/ml vs. 3386 ± 1137 ng/ml, p = 0.004) (Fig. 3A,B).

**Figure 4:**
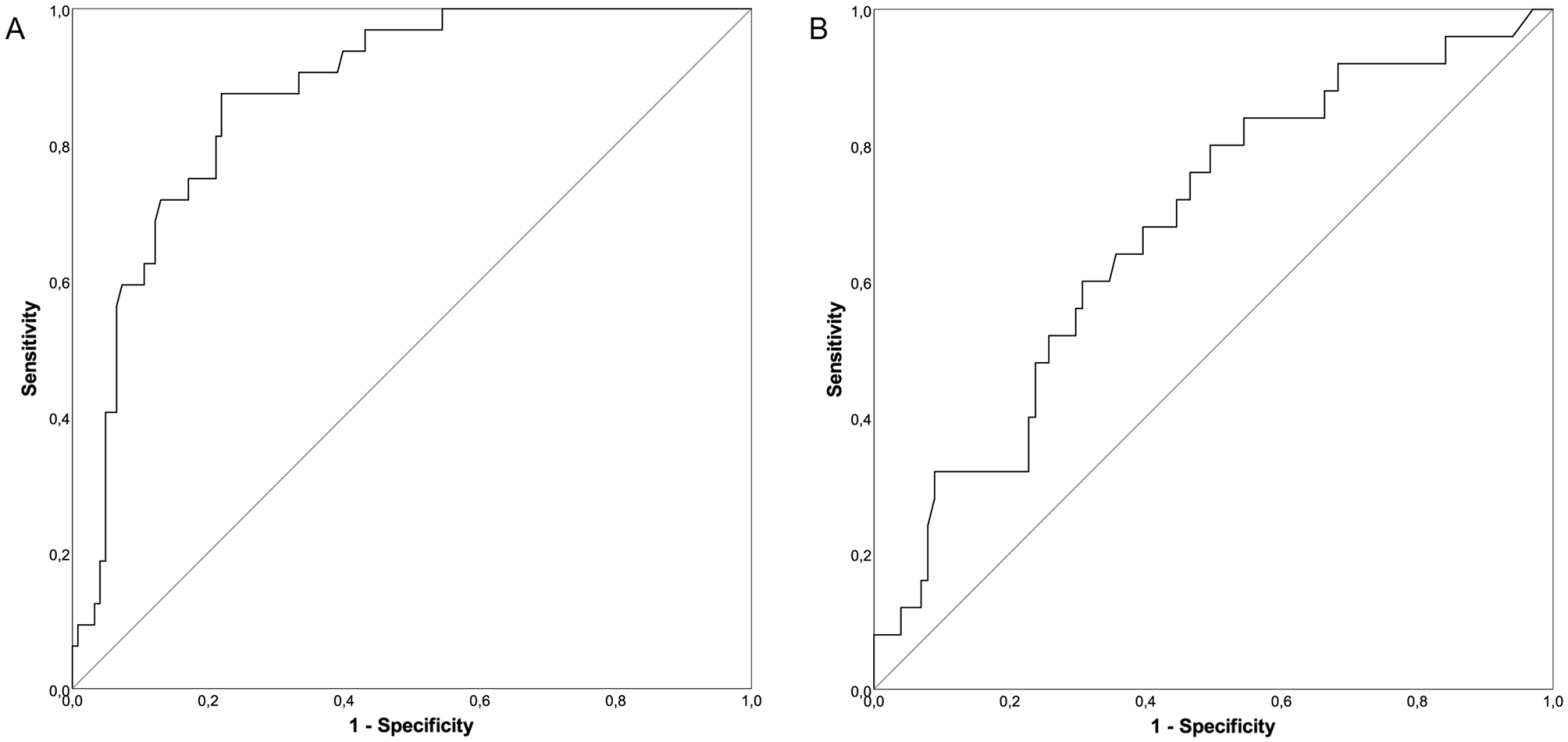
ROC curves of calprotectin levels in serum and saliva. Receiver Operating Characteristic curves to determine cut-off values of calprotectin levels in serum (**A**) and saliva (**B**) using the QUANTUM BLUE sCAL rapid test (black line: ROC curve; grey line: diagonal association line).

The analysis of the QBT levels by ROC curves revealed a cut-off value of 2940 ng/ml (sensitivity = 0.875, specificity = 0.780, area under the curve *A* = 0.873) in serum and 5310 ng/ml (sensitivity = 0.800, specificity = 0.495, *A* = 0.677) in saliva for the existence of a peritonsillar abscess (Fig. 4A,B).

### Adjusted PTA score

The PTA score was developed as an objective screening tool for peritonsillar abscess. This was achieved by taking both characteristic clinical symptoms and increased S100A8/A9 levels in serum and saliva of patients into consideration^14^.

**Figure 5:**
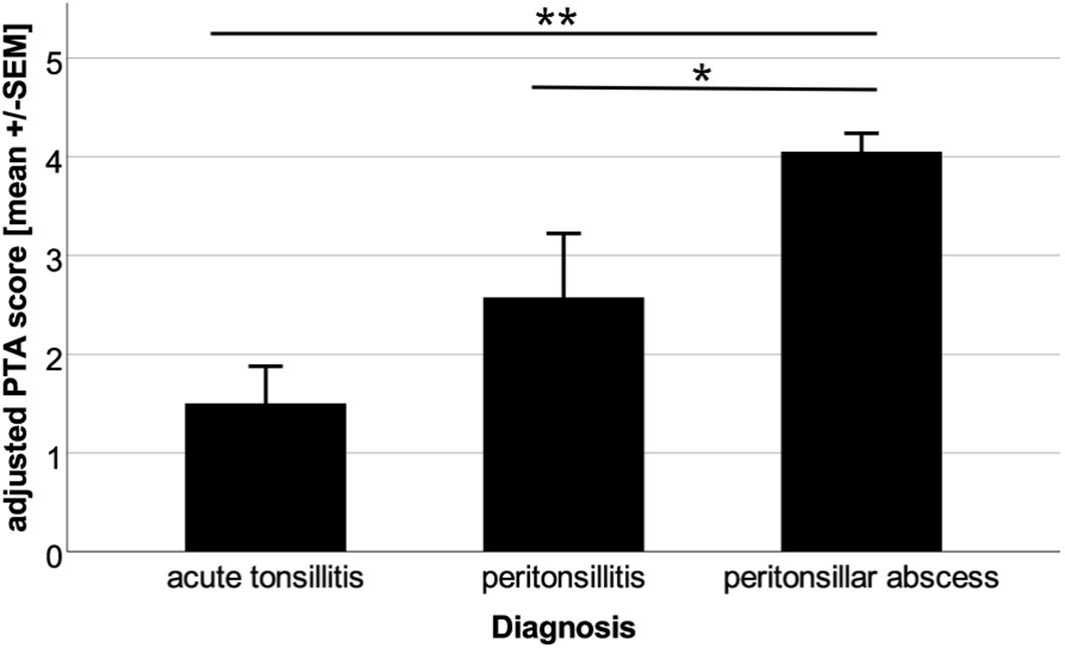
Adjusted PTA score values. Significantly increased adjusted PTA score values could be observed in patients with peritonsillar abscess as compared to peritonsillitis (*p = 0.036) and to acute tonsillitis (**p < 0.001).

**Figure 6:**
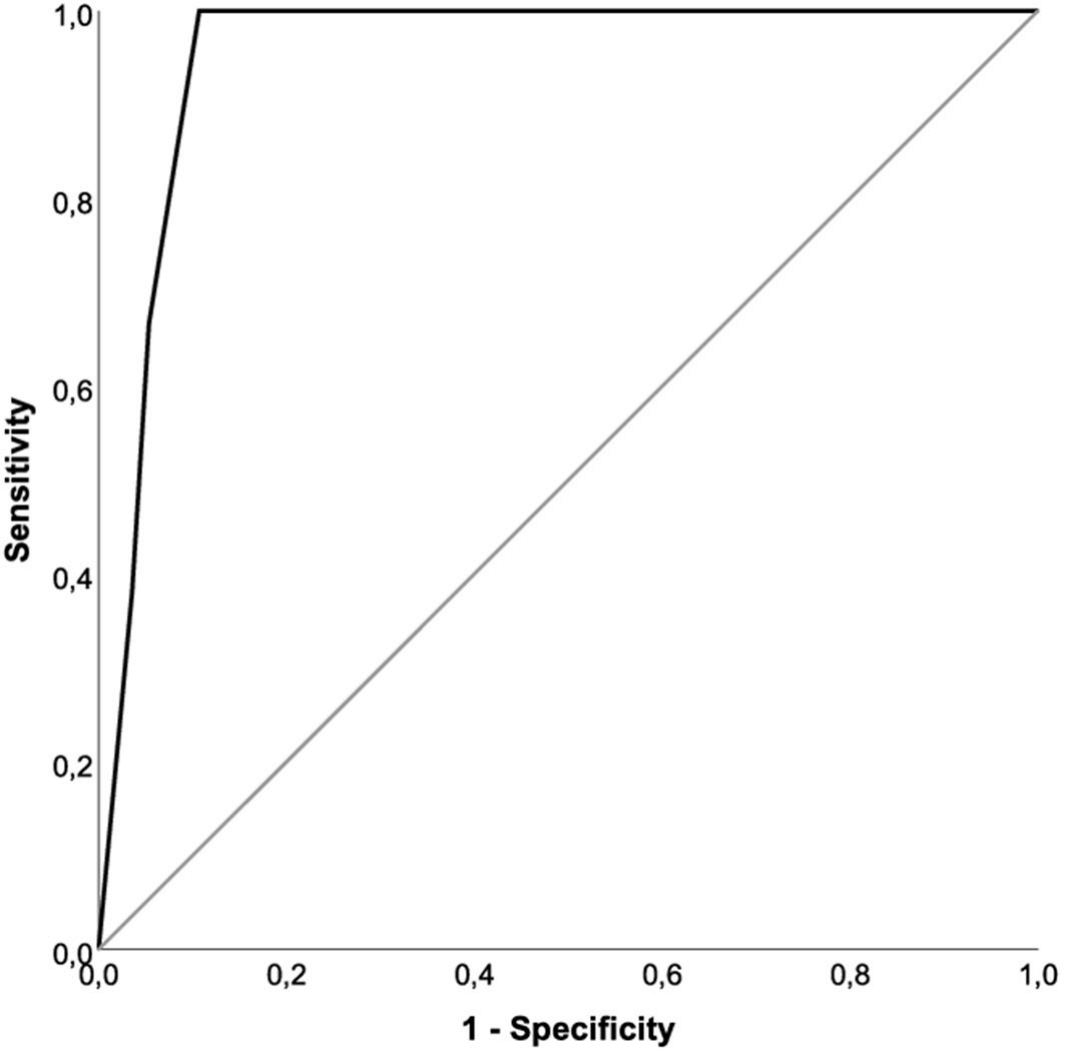
ROC curve of the adjusted PTA score. ROC analysis of the adjusted PTA score revealed a statistical cut-off value of adjPTA = 2.5 (sensitivity = 1.0, specificity = 0.89, p < 0.001) to identify patients suffering from PTA (black line: ROC curve; grey line: diagonal association line).

The PTA score was adjusted based upon the newly determined cut-off values of the calprotectin levels measured by QBT. Hence, one point was added for each clinical symptom and for calprotectin levels above the cut-off values of 2940 ng/ml in serum and 5310 ng/ml in saliva. The clinical symptoms included trismus, halitosis, uvula edema, and unilateral swelling of the arched palate. Patients with PTA showed significantly higher adjusted PTA score values (4.1 ± 0.9, mean ± SEM) compared to patients with acute tonsillitis (1.5 ± 1.1, p < 0.001) and patients with peritonsillitis (2.6 ± 1.7, p = 0.036) (Fig. 5). A ROC analysis of the adjusted PTA score revealed a statistical cut-off value of 2.5 points for the existence of a PTA with a sensitivity of 100% and a specificity of 89.3% (Fig. 6). Since the PTA score from an individual patient is an integer number, a cut-off value of 3 points should be applied in the patient work-up to confirm the existence of a PTA.

## Discussion

Calprotectin highly correlates with the activity of various inflammatory diseases, such as rheumatoid arthritis and inflammatory bowel disease, and was therefore considered a promising and useful biomarker for the differentiation between PTA and PC^10,14,23^. As previously mentioned, a reliable and rapid tool to analyse salivary calprotectin and to differentiate between PTA and PC has not yet been described. POCT methods have become more important over the past years and are useful tools in helping to avoid delays between diagnosis and therapy initiation, especially during outpatient management. Therefore, the aim of this study was to analyse the potential of the QBT as a rapid test in determining calprotectin levels in saliva and serum during outpatient management. To the best of our knowledge, this is the first report describing the QBT as an appropriate POCT tool for quantifying salivary calprotectin levels.

Correlations between salivary calprotectin levels measured by QBT and a “gold standard” ELISA test were assessed in a large cohort of patients with various tonsil-related diseases. This was also done with healthy controls to strengthen the reliability of our findings. Since the QBT was originally developed to determine calprotectin levels in serum samples, we first wanted to verify a positive correlation between the QBT and a “gold standard” ELISA test. Our data impressively demonstrate excellent correlations between the QBT and the BÜHLMANN sCAL ELISA test in saliva (r_sp_ = 0.848). Hence, the QBT is an appropriate tool to determine calprotectin levels in serum and saliva during outpatient management.

In regards to using the QBT in obtaining the PTA score, revised cut-off values for saliva samples may have to be determined due to the slightly positive bias of the QBT as compared to the ELISA. As a result, the PTA score had to be adjusted. Large-scale proteins, such as mucins, may interfere in the lateral flow of the QBT and may be responsible for these differences in salivary calprotectin levels. Another factor potentially hindering a smooth lateral flow through the QBT test cassettes may be the more viscous consistency of saliva as compared to serum. Therefore, the QBT should be further validated and potentially adjusted for saliva measurements, although its agreement to the ELISA is reasonably high. Another critical point is the somewhat limited quantitative measuring range of the QBT as compared to the ELISA. This may lead to increased re-testing of salivary samples, if a precise number is needed. However, by choosing an appropriate dilution of e.g. 1:40 of the salivary samples for the QBT, the recommended cut-off concentration of 5310 ng/ml for diagnosing PTA lies in the middle of the standard curve (in this case from 2000 to 40,000 ng/ml), so that in any case a positive result can be distinguished from a negative one.

By taking the four characteristic clinical symptoms and calprotectin levels in serum and saliva into consideration, the PTA score becomes a powerful tool to identify a PTA. The PTA score has the ability to accomplish this with a sensitivity of 100% and a specificity of 89.3%, respectively. This combination is indispensable, since considering either the symptoms or the calprotectin levels independently would result in a reduction in sensitivity and specificity.

The use of the described POCT tool in combination with the PTA score, especially in the outpatient management of patients suffering from peritonsillar inflammations, reduces the time gap between admission and correct diagnosis. It also helps to determine the appropriate therapy approach, therefore reducing the duration of hospitalisation. More importantly, unnecessary interventions can be avoided, and costs and national health care resources can be reduced^18^.

Our study also proves that calprotectin performs as a good biomarker for differentiation between peritonsillitis and peritonsillar abscess. To confirm the clinical practicability and specificity of the adjusted PTA score, major prospective trials are necessary.

## Conclusion

In summary, the QBT serves as an appropriate POCT tool to determine calprotectin levels in serum and in saliva. In contrast to the ELISA, the QBT is less time-consuming and requires less expertise. Therefore, the QBT can be used well to determine the PTA score values in everyday clinical practice. By combining the QBT and the PTA score, an even faster and more accurate diagnosis of PTA can be achieved during outpatient management.

## Material and methods

### Study design and materials

The study was performed between 2015 and 2017 at the University Hospital Münster, Germany. Serum and saliva samples were gathered from 179 patients with various tonsil-related diseases, such as tonsil hyperplasia, recurrent tonsillitis, acute tonsillitis, mononucleosis, peritonsillar abscess, and peritonsillitis, as well as from healthy volunteers serving as controls (n = 15). Serum samples were collected in aspiration mode using 7.5 ml Serum-Gel Monovette serum tubes (SARSTEDT, Nümbrecht, Germany; order no. 01.1602). Serum was centrifuged at 2000×*g* for 10 min within 2 h after acquisition and the supernatant was aliquoted and stored at − 20 °C until analysis. Saliva acquisition was performed with untreated SALIVETTE (SARSTEDT, Nümbrecht, Germany; order no. 51.1534) as described in the manufacturer’s datasheet or by collecting saliva in a 50 ml Falcon tube (FALCON, 50 ml, conical polypropylene centrifuge tubes) and centrifuged at 1000×*g* for 15 min. Supernatants were aliquoted and stored at − 20 °C until analysis^14^. Patients with malignancy, immunosuppression and pregnancy were excluded. The study was approved by the institutional ethics committee [Ethik-Kommission der Ärztekammer Westfalen-Lippe und der Westfälischen Wilhelms-Universität; 2015-217-f-S]. All research was performed in accordance with ethical principles, including the World Medical Association Declaration of Helsinki (version 2002) and the additional requirements. Written informed consent was obtained from all subjects.

### Enzyme-linked immunosorbent assay (ELISA)

The BÜHLMANN sCAL ELISA (BÜHLMANN Laboratories AG, Switzerland; order no. EK-MRP8/14) was performed according to the manufacturer’s instructions. Briefly, 100 µl of calibrators, controls and previously diluted samples were loaded onto the wells of the microtiter plate which were coated with a monoclonal capture antibody highly specific to the MRP8/14 heterodimeric and heterotetrameric complexes, respectively. After a 30-min incubation at room temperature (18–28 °C) on a rotary shaker and three subsequent washing steps, 100 µl of a monoclonal detection antibody conjugated to horseradish peroxidase (enzyme label) was pipetted to detect the MRP8/14 molecules bound to the monoclonal antibody on the plate in the previous step. After a second 30-min incubation on a rotary shaker and further washing steps, 100 µl of a chromogenic HRP substrate, TMB, was added forming a blue color proportionally to the amount of MRP8/14 present in each well of the microtiter plate. After 15 min on a rotary shaker the color development was stopped with 100 µl of sulfuric acid leading to a color change to yellow. The absorbance of the yellow solution in each well was measured on a microtiter plate reader (Asys Expert 96; Biochrom Ltd., Cambridge, UK) at 450 nm.

This BÜHLMANN sCAL ELISA was successfully used to determine salivary calprotectin levels in periodontitis^24^.

### QUANTUM BLUE sCAL test

The BÜHLMANN QUANTUM BLUE sCAL lateral flow test (QBT; order no. LF-MRP25) was performed according to the manufacturer’s instruction for use. In brief, the samples were diluted in different ratios with chase buffer (from 1:10 up to 1:100) and then 60 µl of the diluted solution was loaded onto the test cassette and incubated for 12 min at room temperature (18–28 °C). After addition of such a diluted serum or saliva sample, a monoclonal anti-MRP14 antibody conjugated to gold colloids deposited onto the conjugate release pad was released onto the test membrane while reacting with the MRP8/14 in the present in the sample. These antibody gold conjugate-MRP8/14 complexes are then bound to a highly specific anti-MRP8/14 antibody deposited onto the membrane (forming a test line). The remaining unbound anti-MRP14 antibody gold conjugate reacted with a goat anti-mouse antibody deposited onto the membrane further downstream (forming a control line). After placing the test cassette in the QUANTUM BLUE Reader, the signal intensities of test and control lines were measured quantitatively by reflectometry, and the reader automatically calculated and displayed the calprotectin values using lot-specific standard curve parameters within seconds.

The QBT was developed and validated for human serum samples with a quantitative measuring range from 0.5 (lower limit of quantitation) to 10 µg/ml (upper limit of quantitation) equalling 500 to 10,000 ng/ml using the recommended sample dilution of 1 in 10^25^. The procedure had to be slightly adapted to account for the expected higher calprotectin levels in saliva of the study population. Therefore, the saliva samples were diluted 1 in 40 with chase buffer. If still above the upper limit of quantitation, a second sample was re-measured applying a 1 in 100 dilution with chase buffer. The effective salivary calprotectin concentrations were adjusted by these additional dilution factors. In this way, a quantitative measuring range from 2000 up to 100,000 ng/ml could be achieved for salivary calprotectin.

### PTA score

The PTA score was developed in order to differentiate between a peritonsillar abscess and peritonsillar cellulitis as described elsewhere^14,18^. Symptoms such as halitosis, trismus, uvula edema and unilateral swelling of the arched palate represent characteristic clinical symptoms of peritonsillar abscess and are used to determine the PTA score. For the presence of each of the four clinical symptoms and S100A8/A9 values in serum and/or saliva above the cut-off level(s), one point is added to the PTA score, which results in a range from a minimum of zero points to a maximum of six points. Higher scores indicate a higher probability of the existence of a peritonsillar abscess. The survey examining the presence of symptoms and the rapid screening test for calprotectin in serum and saliva appear to be essential for the appropriate diagnosis of PTA^14^. In concordance with the determined cut-off values for calprotectin levels in serum and saliva, we adjusted this PTA score using the QBT results (adjPTA).

### Statistical analysis

Descriptive results were reported as mean with onefold standard error of the mean (SEM). Strengths of the monotonic association between non-parametric variables were determined by Spearman’s correlation coefficients (r_sp_) and were considered to be low (0.2 < r_sp_ ≤ 0.5), good (0.5 < r_sp_ ≤ 0.8) or excellent (r_sp_ > 0.8). A Bland–Altmann analysis was performed to determine the agreement of salivary calprotectin levels between QBT and ELISA procedures^26^. Receiver-operating-characteristic (ROC) curves were determined to illustrate the capacity of a model to differentiate between positive and negative results. Furthermore, cut-off values and area under the curve values (*A*-values) were determined to analyse the discriminative power that can be described as excellent with an *A*-value of > 0.9, good > 0.8, acceptable > 0.7 or poor < 0.7. The Mann–Whitney U-test was performed to determine differences between independent, non-parametric data. Results with p < 0.05 were considered to be statistically significant. Data were collected and analysed using IBM SPSS Statistics 25 for Windows (IBM Corporation, Somers, NY, USA). The investigator was blinded during the entire testing period for diagnosis. Statistical advice was given by the Institute of Biostatistics and Clinical Research, University of Münster, Germany.

### Ethics approval and consent to participate

The study was approved by the institutional ethics committee [Ethik-Kommission der Ärztekammer Westfalen-Lippe und der Westfälischen Wilhelms-Universität; 2015-217-f-S] and written informed consent was obtained from all subjects.

## Data Availability

The datasets used and/or analysed during the current study are available from the corresponding author on reasonable request.
